# Severe Nonspecific Interstitial Pneumonia (NSIP) in an Adolescent

**DOI:** 10.1155/2022/7757776

**Published:** 2022-08-09

**Authors:** Giuliana Cerro Chiang, Christopher Lee, Alberto Marchevsky, Michael I. Lewis

**Affiliations:** ^1^Division of Pulmonary and Critical Care Medicine, Department of Medicine, Cedars-Sinai Medical Center, Los Angeles CA, USA; ^2^Department of Radiology, Cedars-Sinai Medical Center, Los Angeles CA, USA; ^3^Department of Pathology, Cedars-Sinai Medical Center, Los Angeles CA, USA

## Abstract

Childhood interstitial lung disease (chILD) is remarkably rare with a reported prevalence from 0.13 per 100,000 children under 17 years to 16.2 per 100,000 children under 15 years of age (Kornum et al., 2008). Here, we present a case of a 15-year-old with subacute hypoxemic respiratory failure, admitted to the critical care unit. Her imaging on admission showed bilateral interstitial infiltrates; her laboratory workup, including autoimmune serologies, was unrevealing. A bronchoscopy revealed the diagnosis of nonspecific interstitial pneumonia. She had a partial recovery after a course of steroids.

## 1. Introduction

Childhood interstitial lung disease (chILD) is rare with reported prevalence from 0.13 per 100 000 children under 17 years to 16.2 per 100 000 children under 15 years of age [1, 2]. chILD can be classified into 4 groups: ILD related to exposures or environmental insults, ILD related to systemic disease processes, ILD related to primary lung parenchyma dysfunctions, and ILD specific to infancy [3]. Among the interstitial pneumonias in childhood, nonspecific interstitial pneumonia (NSIP) is exceedingly rare, and its occurrence has mostly been described in single case reports [4]. Here, we describe an unusual case of NSIP in a 15-year-old adolescent who presented in acute on chronic hypoxemic respiratory failure whose diagnosis was verified by surgical lung biopsy and responded to immunosuppressive treatment.

## 2. Case Report

### 2.1. History and Physical Examination

A 15-year-old female with no known past medical history presented with a 3-week history of progressive dry cough, fever, malaise, and dyspnea on exertion. Her symptoms had progressed to the point that she was unable to ambulate around her home. Her fever was intermittent and as high as 102°F (38.9°C). Prior to admission, she received a course of azithromycin and acetaminophen with no improvement.

She had no history of travel. She had two parakeets 8 years prior to presentation but currently no pets. She denied any alcohol, tobacco, vaping, or other drug use and had no known mold exposures. Family history was notable for an aunt with rheumatoid arthritis as well as uncles with asthma.

On physical examination, she was normotensive, afebrile, tachycardic, and tachypneic. Her oxygen saturation was 80% on room air; she was unable to speak full sentences due to dyspnea. No signs of congestive heart failure were evident. Cardiac examination was within normal limits. Auscultation of the chest revealed coarse breath sounds throughout her lung fields. There were no signs of respiratory loading (supraclavicular retraction accessory respiratory muscle use or abdominal paradox). Abdominal examination was unrevealing. Dermatologic and musculoskeletal examinations demonstrated no rashes, arthritis, or synovitis. No neurologic signs were evident.

### 2.2. Workup

Initial labs showed a normal complete blood count including a normal cell differential. The basic metabolic panel and liver function tests were normal. She had negative cardiac markers. C-reactive protein was elevated (65.7 mg/L) as well as sedimentation rate (92 mm/h). Ferritin and IL-6 were also elevated at 246 ng/mL and 18 pg/mL, respectively.

She had a negative respiratory viral and atypical pathogen PCR panel including two negative SARS-CoV-2 PCRs and serologies. Further infectious workup including bacterial and fungal blood cultures, HIV, and fungal serologies was unrevealing. Comprehensive autoimmune serologies were notable for elevated ANA at a 1 : 80 titer with a nucleolar pattern. Her IgE was elevated (762 kU/L), but she did not have any eosinophilia. Additional autoimmune serologies, myositis panel, and hypersensitivity pneumonitis panel were all negative. Her spirometry showed a FVC 0.84 L (26% predicted), FEV1 0.80 L (25% predicted), FEV1/FVC ratio 86%, TLC 1.62 L (38% predicted), and DLCO 7 (31% predicted) consistent with a severe restrictive ventilatory defect. She underwent bronchoscopy with transbronchial biopsies of the right lower lobe, followed about 3 weeks later by video-assisted thoracoscopy surgery (VATS) with wedge lung biopsies of the right middle and lower lobes.

### 2.3. Radiologic Findings

**Figure 1:**
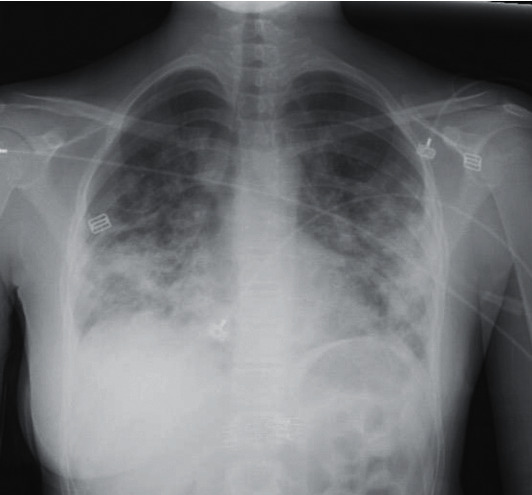
Admission chest radiograph demonstrates bilateral ill-defined opacities with mid and lower lung predominance.

**Figure 2:**
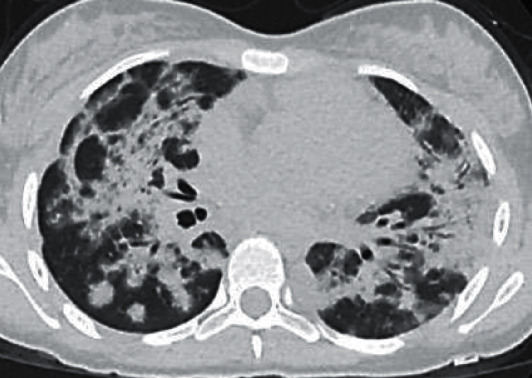
Axial high-resolution CT demonstrates extensive bilateral peribronchovascular consolidations with air bronchograms, as well as scattered small nodular opacities.

**Figure 3:**
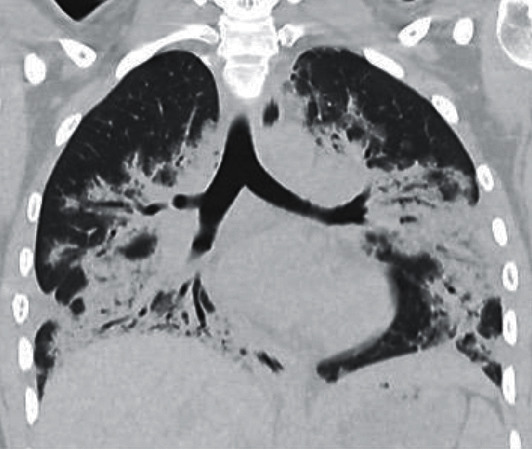
Coronal high-resolution CT image demonstrates extensive bilateral peribronchovascular consolidations with air bronchograms, as well as scattered small nodular opacities.

Chest radiography on admission showed bilateral basilar interstitial infiltrates and confluent consolidations (Figure 1). An initial CT of the chest showed diffuse bilateral consolidations with central predominance and apical sparring. These consolidations did not spare the pleural space, and there were no traction bronchiectasis, fibrotic changes, or pleural effusions (Figures 2 and 3). The differential diagnosis included organizing pneumonia, viral pneumonia with acute lung injury, or connective tissue disease ILD.

### 2.4. Pathology Findings

**Figure 4:**
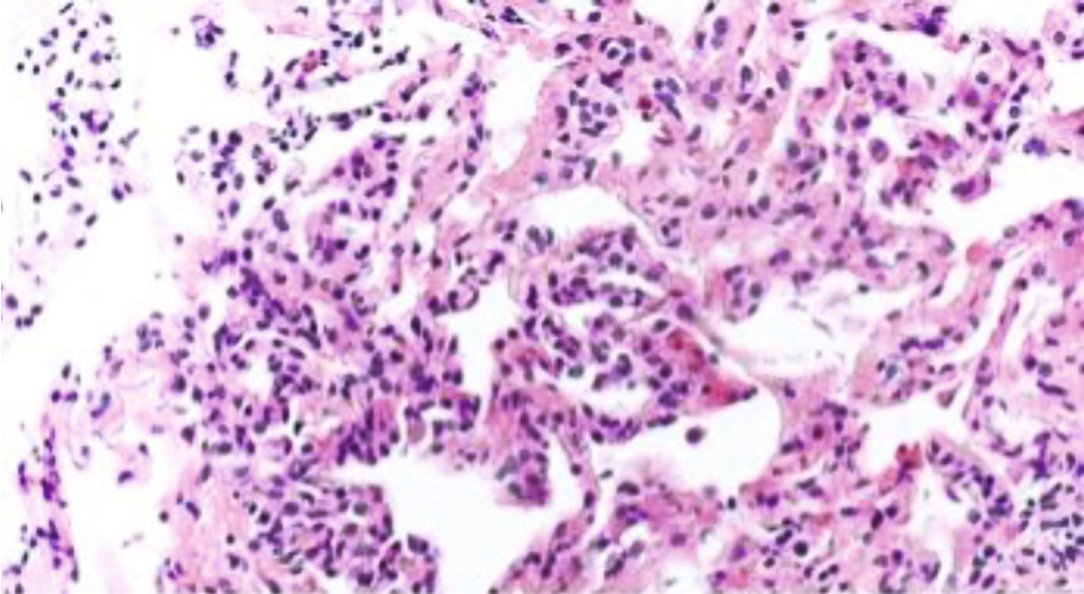
Photomicrograph of transbronchial biopsy showing mild lymphocytic alveolar infiltrates (hematoxylin and eosin 200x).

**Figure 5:**
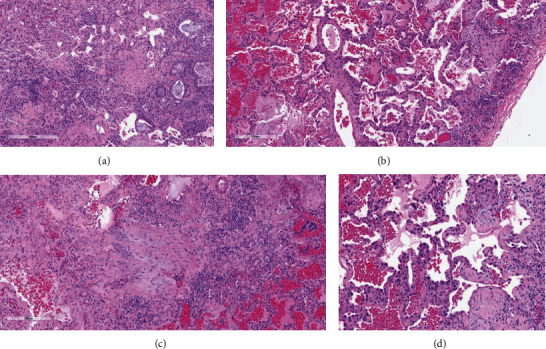
(a) Photomicrograph of the wedge biopsy from the lower lobe showing interstitial lymphocytic alveolar infiltrates and adjacent interstitial fibrosis and peribronchiolar metaplasia (hematoxylin and eosin 20x). (b) The lower lobe wedge biopsy also showed areas with mild, diffuse interstitial fibrosis characteristic of fibrotic nonspecific interstitial pneumonia (NSIP) pattern of fibrosis (hematoxylin and eosin 20x). (c) The wedge biopsies showed only focal areas of organizing pneumonia with early intraalveolar fibrosis involving less than 10% of the tissues (hematoxylin and eosin 20x). (d) Foci of organizing pneumonia (hematoxylin and eosin 20x).

**Figure 6:**
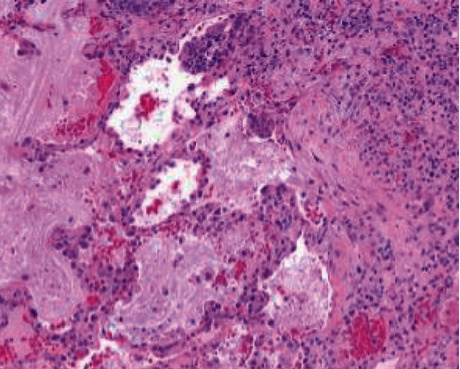
Isolated fibroblastic foci (hematoxylin and eosin 100x).

The transbronchial biopsies showed a mild interstitial pneumonia with minimal interstitial fibrosis (Figure 4). The differential diagnosis included an atypical pneumonia, cellular nonspecific interstitial pneumonia (NSIP), and hypersensitivity pneumonitis. Due to persistent hypoxemia and a lack of a clear diagnosis, she underwent video-assisted thoracoscopy surgery (VATS) with lung biopsy. Both wedge lung biopsies showed a diffuse chronic interstitial pneumonia with scattered lymphoid infiltrates of alveolar spaces and mild interstitial fibrosis (Figures 5(a) and 5(b)). Focal areas of organizing pneumonia and subpleural foci of peribronchiolar metaplasia with microscopic honeycombing were also present (Figures 5(c) and 5(d)). There was a scattered fibroblastic foci (Figure 6). No granulomas or findings consistent with vasculitis were seen. The histopathologic diagnosis nonspecific interstitial pneumonia with fibrosing pattern.

### 2.5. Management

Her case was discussed in a multidisciplinary interstitial lung disease meeting, and based on the radiological and pathology findings, a diagnosis of NSIP of unknown etiology was made. She was discharged on a slow taper of prednisone and referred to pulmonary rehabilitation and has continued to improve clinically.

## 3. Discussion

Nonspecific interstitial pneumonia (NSIP) is a rare interstitial lung disease. Initially described as a pattern of intestinal pneumonitis of varying etiologies, it was accepted in 2013 as a distinct entity in the American Thoracic Society, International Multidisciplinary Classification of Interstitial Pneumonias [5].

Possible causes of NSIP include connective tissue disease, hypersensitivity pneumonitis, drug toxicities, and human immunodeficiency virus. Idiopathic NSIP is a diagnosis of exclusion [6].

NSIP in children is rare. A case series from Nicholson et al. in 1998 found seven cases of NSIP among 25 children who underwent surgical lung biopsies based on the absence of classic features for other entities such as lymphoid interstitial pneumonia, follicular bronchiolitis, chronic pneumonitis of infancy, and desquamative interstitial pneumonitis [7].

Radiologically, the most common abnormality is pleural sparring and bilateral ground glass opacities with traction bronchiectasis and bronchiolectasis. Consolidation may reflect a component of organizing pneumonia or connective tissue disease [8].

Histologically, NSIP is characteristic for temporal uniformity and inflammation and fibrosis of the alveolar walls. The pathologic infiltrate is characterized by lymphocytes and plasma cells. If fibrosis is present, it appears as collagen bundles with few fibroblasts [6]. Notably, few focal areas of organizing pneumonia can be seen as well as rare fibroblastic foci. Patients with a predominantly cellular pattern have a better response to immunosuppressants than those who have a fibrotic pattern.

The patient described in the case did not have a classic radiologic pattern of NSIP given the predominance of consolidation; however, this diagnosis was confirmed by surgical lung biopsy. Notably, this was obtained after a course of steroids; so presumably, she had a flare of her disease which appeared as organizing pneumonia which improved by the time tissue sampling was obtained.

In our case, the samples obtained by transbronchial biopsies were not sufficient to make a definite diagnosis. Compared to surgical lung biopsy, transbronchial forceps biopsy can provide enough information to diagnose about 20-30% of the patients with ILD [9]. One of the advantages is less invasive with minimal rate of complications, namely, pneumothorax, compared to surgical lung biopsy. In the latest idiopathic pulmonary fibrosis guidelines, transbronchial cryobiopsy has emerged as an acceptable alternative to surgical lung biopsy, with similar diagnostic yield and lower rate of complications [10].

In terms of possible etiologies, she had a mildly elevated antinuclear antibody titer with remaining autoimmune serologies. Interstitial pneumonia with autoimmune features (IPAF) is a research diagnosis in patients with interstitial lung disease who have some features of autoimmune diseases. Ademhan described a cohort of children with ILD in which 4.5% of the cases were classified as IPAF, all of them present in female teenagers [11]. Our patient did not meet criteria for IPAF based on the absence of clinical features and a ANA titer below the diagnostic cutoff of 1 : 320.

Another consideration was hypersensitivity pneumonitis, which can also mimic NSIP histologically with the presence of poorly formed granulomas, but the patient had no exposures.

Other etiologies such as HIV, drugs, and toxins have also been associated with NSIP; our patient had a negative HIV test and no drug or toxin exposures. Finally, cigarette smoking and vaping are also associated with NSIP, but our patient had neither.

### 3.1. Therapy

Corticosteroids are the initial agent of choice. Other immunosuppressive medications such as azathioprine and mycophenolate of cyclophosphamide can be used as steroid sparring agents. The response of treatment is variable [12]. Patient with predominance of the cellular subtype tends to have a better response than those with the fibrotic subtype [13, 14]. Our patient had a partial response to corticosteroids early on in her treatment, manifested by improved gas exchange and improved symptoms. The relatively slow response to treatment in the first several weeks is likely consistent with her biopsy findings which showed fibroblast proliferation and fibrosis.

## Data Availability

No data were used to support this study.
